# Playing *Minecraft* Improves Hippocampal-Associated Memory for Details in Middle Aged Adults

**DOI:** 10.3389/fspor.2021.685286

**Published:** 2021-07-05

**Authors:** Craig E. L. Stark, Gregory D. Clemenson, Ujwal Aluru, Nikki Hatamian, Shauna M. Stark

**Affiliations:** Department of Neurobiology and Behavior, University of California, Irvine, Irvine, CA, United States

**Keywords:** video game, memory, hippocampus, Esports, middle age

## Abstract

Concerns are often raised about the impact that playing video games may have on cognition and behavior, whether gameplay is intense and protracted as with competitive Esports or whether it is more casual gameplay. Work in our lab and others, however, has shown that at least some classes of games can improve memory function. In particular, playing immersive 3D games that provide rich experiences and novelty improve memory on tasks that rely upon the hippocampus in effects that mirror the effects of “environmental enrichment” in numerous rodent studies. Our goal in the present study was to determine whether even modest amounts of gameplay (~30 min/day for 4 weeks) would result in improved memory performance in middle-aged adults. Not only is this demographic potentially highly receptive to gaming (they make up a significant portion of Esports viewers and of game players), but interventions in middle age may be a prime time for reducing later age-related cognitive decline. Here, we found that the benefits in middle age paralleled effects previously observed in young adults as playing *Minecraft*, showing improved memory performance on a hippocampal dependent memory task.

## Introduction

Video games and Esports do not always enjoy a positive view in our society, despite the popularity of video games and the rising impact of Esports (Duggan, 2015). Yet, at their core, modern video games often provide incredibly rich cognitive experiences, opportunities for problem solving, for competition, for teamwork, and for social interaction. As a result, gaming and Esports have the potential to provide considerable positive effects to the brain.

One clear aspect of many modern video games is that they provide a novel, rich world to explore. Decades of neuroscience research dating back to pioneering efforts by Donald Hebb (Hebb, 1947) have shown that simply placing laboratory animals in enriched environments improves cognitive performance within a wide range of underlying neurobiological mechanisms [Clemenson et al. (2018) and Kempermann (2019) for review]. This “environmental enrichment” can not only ameliorate age-related effects on memory and structures in the brain like the hippocampus, known to support memory (Kempermann et al., 2002; van Praag et al., 2005; Segovia et al., 2006; Speisman et al., 2013), but it has been shown to reduce the presence of both the amyloid-beta plaques and the neurofibrillary tangles in mouse models of Alzheimer's Disease (Lazarov et al., 2005; Hu et al., 2010, 2013), rescue deficits in hippocampal neurogenesis and synaptic plasticity (Hu et al., 2010; Rodríguez and Verkhratsky, 2011; Valero et al., 2011), improve hippocampal-based memory (Arendash et al., 2004; Jankowsky et al., 2005), and up-regulate neurotrophic factors important to environmental enrichment and hippocampal neurogenesis (Wolf et al., 2006; Hu et al., 2010). Thus, there is substantial neurobiological evidence to support the idea that environmental enrichment, even from video games, might have a positive effect on the hippocampus and the memory abilities it supports.

Prior research in our lab and others has demonstrated positive effects of large, immersive 3D video game playing on hippocampal-based memory ability (Clemenson and Stark, 2015; Clemenson et al., 2019, 2020; Wais et al., 2021). For example, in an intervention using young non-gamer adults, we found that 2 weeks of playing *Super Mario 3D World* improved hippocampal-based memory performance relative to both active and no-contact control groups (Clemenson and Stark, 2015), with the amount of exploration in the game correlating with the amount of improvement. We found a similar effect in older adults (60–80 years) with 4 weeks of playing improving memory ability such that it matched performance of participants 15–20 years younger (Clemenson et al., 2020). In yet another study, we used *Minecraft* to more directly manipulate the amount and type of enrichment (Clemenson et al., 2019). In this study, 2-weeks of spatial exploration of the virtual world and building complex structures with resources gathered in the world resulted in a robust improvement in memory ability. In contrast, unconstrained building in a world devoid of spatial features (“free building”) showed no effect on memory ability. Having participants learn to build complex structures in a flat world (“directed building”) or having them merely explore the world (“exploration”) both involved substantial enrichment and both resulted in strong effects (albeit seemingly less robust than the combination of the two).

Our goal in the present study was to determine whether a similar intervention using *Minecraft* would benefit middle-aged adults. If environmental enrichment *via* video games can improve memory, interventions in middle-age may be more impactful and viable than interventions in older adults. Recent statistics show that the average age of Esports fans is 32 and the average age of gamers is 37, with roughly a third of all gamers surveyed being between 35 and 55 (Nielsen, 2015), indicating that this age range may be more likely to adopt video game playing as a routine activity. In addition, an intervention before the onset of substantial cognitive decline may be more effective than after decline has set in. Here, *Minecraft* provided us a way to deliver a rich, immersive experience previously shown effective in young adults that also would be approachable for non-gamers whose last experience with gaming may-well have dated back several decades.

## Materials and Methods

We studied three main groups of participants: no-contact control (CON-NC), active-control (CON-Active), and Intervention. All groups received the same cognitive testing, consisting of an online version of the Mnemonic Similarity Task (MST; Kirwan and Stark, 2007; Stark et al., 2019) administered within 1.5 weeks of beginning any intervention and of completing any intervention. In addition, the CON-Active and Intervention group played *Minecraft* for ~30 min per day during the 4 weeks of the experiment on their own computer. Participants were directed to individual servers housed in our lab with customized worlds and instructed not to play in other *Minecraft* worlds on their own during the course of the experiment. Log-files were monitored to ensure that they kept up with ~30 min / day for 5-days each week during the experiment.

Based on our prior study (Clemenson et al., 2019), we created an active control condition for the CON-Active group that used an open-ended “free building” environment in which participants were placed in a barren world, but given a box of infinite resources and instructed to build whatever they wanted. Also based on our prior study (Clemenson et al., 2019), we created two different active intervention environments for the Intervention group. In one (“exploration”), participants were placed in a typical Minecraft world, given instructions to explore, and to return to their starting, “home” location at the end of each session. In the second (“directed building”), participants were placed in a barren world and given instructions on how to build increasingly more complex and difficult structures, along with a box that contained the required resources. These two conditions yielded virtually identical results in young adults in our prior work and are less taxing than the “explore and build” group used previously. Thus, we included both conditions here to allow for exploratory analyses in this middle-aged group, either to replicate the results in young adults or to serve as an interesting difference across age. Given that both conditions yielded a similar benefit, our primary analyses collapse across these conditions. We chose not to include our prior “explore and build” to limit the complexity of the design and the demands on participants who were performing the intervention at home.

### Participants

Ninety-two participants were recruited from the surrounding community to participate in the study using a combination of e-mail advertising and University of California, Irvine's Consent to Contact database (Grill et al., 2018). Participants were required to be between the ages of 40–49, be proficient in English, have no self-reported history of neurological damage or drug abuse, and not routinely play PC/Console based video games (occasional play of mobile games allowed). In addition, we used the Montreal Cognitive Assessment (Nasreddine et al., 2005) and a threshold of 26 during screening to identify and exclude any potential participants with apparent cognitive disfunction. Participants were assigned pseudo-randomly to conditions. We targeted an *n* = 30 in our CON-NC control condition, an *n* = 20 for our CON-Active control, and an *n* = 40 for our Intervention condition (split evenly between “directed building” and “exploration”). We chose these sample sizes for several reasons. First, our primary outcome measure would be based on the contrast between an *N* = 40 intervention and an *N* = 30 control group. Based on our prior work, these sample sizes would result in more than enough statistical power (where *N*'s of 20–30 were appropriate). While our two intervention groups might have been run at *N* = 15 each, we wished to have the option to perform reasonable exploratory analyses and reasoned that this would likely be insufficient and/or too risky given challenges associated with dropout or data loss. Thus, we balanced resources (time and funding) and our desire to maximize our scientific potential in deriving these target numbers. Throughout the experiment, if participants did not engage with the intervention or at-home testing, they were removed and we attempted to assign new participants to their condition. Given the long timescale of the study and given the fact that performance could be used to eventually remove a subject from analysis (see Results), the dynamics of the study has us over-enroll the control conditions slightly (31/30 and 22/20). In the final analysis, we had 30 CON-NC (21 female, average age 43.5), 21 CON-Active (11 female, average age 45.5), and 35 Intervention participants (17 female, average age 45.9).

### Minecraft

Minecraft is an open-world, sandbox video game in which players typically explore a procedurally generated world consisting of different biomes (plains, jungles, deserts, forests, mountains, etc.). In the game, players can harvest materials and use these to build complex structures. In the experiment, all worlds were created ahead of time to suit the various experimental conditions (one consistent map per condition) and each participant inhabited a personal, customized world hosted on a server in our lab. Custom servers were created using SpigotMC (www.spigotmc.org) and a custom mod, Tracker (created by GDC), was added to track spatial locations of individuals and information about the world they created. The Minecraft world and spawn locations were the same for every individual, ensuring that all participants received the same starting experience. The difficulty for all servers was set to “peaceful,” which removed enemies.

Prior to gameplay, participants installed *Minecraft* on their own computers (or, in several cases, computers loaned to the participants) with our assistance and they were given ~30 min of instruction on basic gameplay and on their particular tasks. The active control condition consisted of the same “free building” condition used in our prior work (Clemenson et al., 2019). Here, worlds were devoid of all features (no biomes, mountains, animals, etc.) but participants were given chests full of blocks and instructed to build whatever they wished. In the “directed building” sub-group of the Intervention condition, the worlds were the same except for the contents of the chests. Here, they provided just enough resources to build specific structures (blueprints taken from www.grabcraft.com) assigned to participants. In the 1st week, participants were assigned specific structures to ensure they understood the controls and mechanics of *Minecraft* and then they were allowed free choice of structure. Finally, in the “exploration” sub-group of the intervention condition, participants played *Minecraft* as it is typically played. They spawned in at the exact same location on the same map, and were instructed to first build some form of “home.” After that, they were free to play however they saw fit with the only constraint that they were to return to the starting location at the end of each session.

### Online Cognitive Testing With the MST

Our lab developed the MST as a robust test of memory performance (Kirwan and Stark, 2007; Stark et al., 2019) and has made numerous versions of it available on our website and on GitHub. Here, we created a version of the MST in JavaScript using the jsPsych library for web-based deployment (de Leeuw, 2015) to allow for remote testing. It is a mature, free, stable library that has been rigorously tested for even demanding reaction time-based experiments (de Leeuw and Motz, 2016; Hilbig, 2016; Pinet et al., 2017). In addition, we integrated the task with the open-source JATOS package (Lange et al., 2015) to provide a reliable means of securely administering test sessions on the web and managing the data.

**Figure 1:**
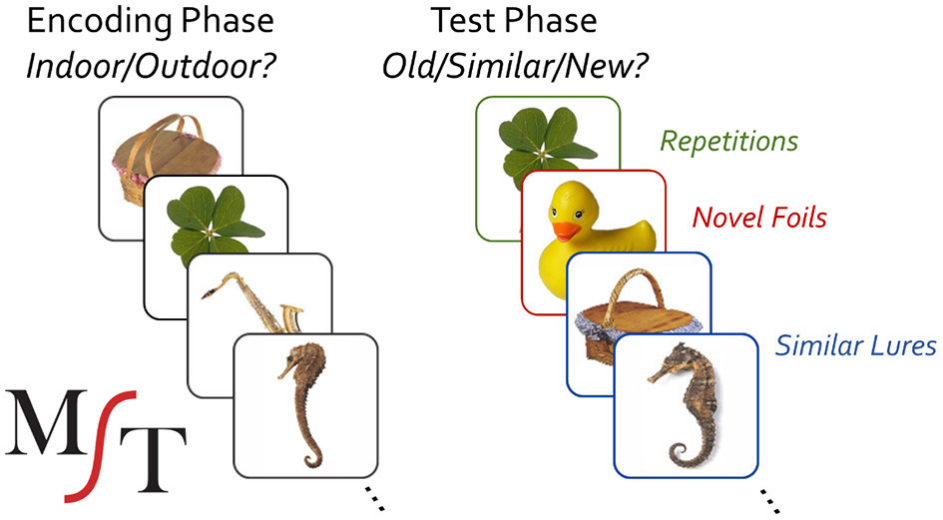
Mnemonic similarity task; colored boxes are to illustrate conditions, but not used during the actual task administration.

The MST has been extensively described elsewhere (Stark et al., 2013, 2019). Briefly, it consists of a traditional object-recognition memory task, modified to include highly similar lure items that tax pattern separation and hippocampal function (Figure 1). It typically consists of separate incidental encoding and explicit test phases. During encoding, participants are shown 128 color pictures of everyday objects (2 s duration, 0.5 s ISI) and asked to indicate whether they are typically indoor or outdoor objects. A brief video instruction precedes the task to show them sample trials and to let them know there is no “correct” answer to this question. In a surprise recognition test, participants are then instructed to make a 3-choice old/similar/new judgment for three types of trials: repeats, lure items that are similar but not identical to study items, and novel foils. Stimuli are shown for 2 s at test (0.5 s ISI), but participants can take as much time as needed to make their response (i.e., the ISI is potentially infinite as the experiment will await responses with no object shown). A total of 192 images are shown at test with 64 items being repeated from study, 64 being highly similar lures, and 64 being novel foils.

The MST produces two output measures based on memory performance: LDI and REC. Its LDI or “Lure Discrimination Index” measure reflects the probability that participants can successfully determine that similar lure items (e.g., the seahorse or picnic basket) are not, in fact, repetitions of the studied item and is computed by *p*(“Similar”|Lure)—*p*(“Similar”|Foil). The REC measure is a traditional object recognition memory measure, reflecting the probability of endorsing true repetitions (e.g., the clover) vs. incorrectly endorsing unrelated foils (e.g., the rubber duck) and is computed by *p*(“Old”|Repetition)–*p*(“Old”|Foil).

### Data Analysis

Data quality control standards were applied to all conditions equally. To be included, participants must have completed at least 3 weeks of any intervention and been tested within 1.5 weeks of the target end-date. In addition, we used a 50% correct threshold on the traditional corrected object recognition memory score (REC) from the MST to ensure participants were actively engaged in testing (the REC score is not a primary outcome measure and should not change as a result of the intervention). Finally, we required that LDI scores not be negative as this would indicate either a lack of understanding of the instructions or clearly chance performance. Invalid data in either the pre- or post-test invalidated all data from that participant.

## Results

Of the 31 participants assigned to the CON-NC condition, one was dropped for exceptionally poor REC performance (9% correct). Of the 22 participants in the CON-Active condition, one was dropped for failing to complete the study. Of the 40 participants in the active Intervention group, five were dropped for failing to complete, two for exceptionally poor REC performance (44 and 17%), and two for strongly negative LDI scores (−0.44 and −0.19).

Following this data quality screening, we examined baseline LDI and REC performance in each group (Table 1). All three groups began with similar LDI [CON-NC = 0.38 ± 0.22, CON-Active = 0.36 ± 0.18, Intervention = 0.36 ± 0.13; one-way ANOVA *F*_(3, 80)_ = 0.10, *p* = 0.90] and similar REC [CON-NC = 0.81 ± 0.11, CON-Active = 0.82 ± 0.12, Intervention = 0.83 ± 0.09; one-way ANOVA *F*_(3, 80)_ = 0.33, *p* = 0.72] scores on the MST, indicating there were no differences in baseline performance across groups. In addition, we compared the average (Intervention: 38 min/day ± 19 min; CON-Active: 38 min/day ± 16 min) and total play time (Intervention: 764 min ± 322 min; CON-Active: 785 min ± 386 min) across groups. While there was clearly substantial variance across participants, the groups did not differ (unpaired *t*-test *p*'s > 0.8).

**Table 1:** Pre- and post- response rates, LDI and REC scores.

	**CON-NC**		**CON-Active**		**Intervention**	
	**Mean**	**SD**	**Mean**	**SD**	**Mean**	**SD**
Pre-RO	0.819	0.113	0.836	0.125	0.846	0.081
Pre-RS	0.143	0.093	0.125	0.104	0.118	0.066
Pre-RN	0.034	0.034	0.042	0.055	0.038	0.038
Pre-LO	0.398	0.184	0.407	0.145	0.428	0.106
Pre-LS	0.500	0.184	0.513	0.186	0.491	0.129
Pre-LN	0.092	0.065	0.083	0.071	0.082	0.058
Pre-FO	0.019	0.017	0.018	0.015	0.020	0.032
Pre-FS	0.129	0.079	0.152	0.083	0.135	0.088
Pre-FN	0.847	0.087	0.832	0.089	0.847	0.094
Pre-REC	0.805	0.113	0.818	0.128	0.828	0.094
Pre-LDI	0.376	0.216	0.359	0.182	0.356	0.128
Post-RO	0.773	0.192	0.806	0.107	0.772	0.138
Post-RS	0.138	0.114	0.153	0.075	0.181	0.127
Post-RN	0.044	0.057	0.043	0.047	0.040	0.041
Post-LO	0.332	0.204	0.304	0.159	0.277	0.140
Post-LS	0.527	0.206	0.615	0.188	0.636	0.157
Post-LN	0.095	0.075	0.083	0.070	0.083	0.056
Post-FO	0.020	0.036	0.022	0.028	0.019	0.024
Post-FS	0.110	0.074	0.158	0.071	0.137	0.085
Post-FN	0.820	0.188	0.822	0.080	0.843	0.096
Post-REC	0.794	0.156	0.785	0.118	0.756	0.142
Post-LDI	0.441	0.228	0.456	0.203	0.506	0.178

*R, Repeat; L, Lure; F, Novel foil; O, ”Old” response; “S”, Similar response; “N”, New response*.

**Figure 2:**
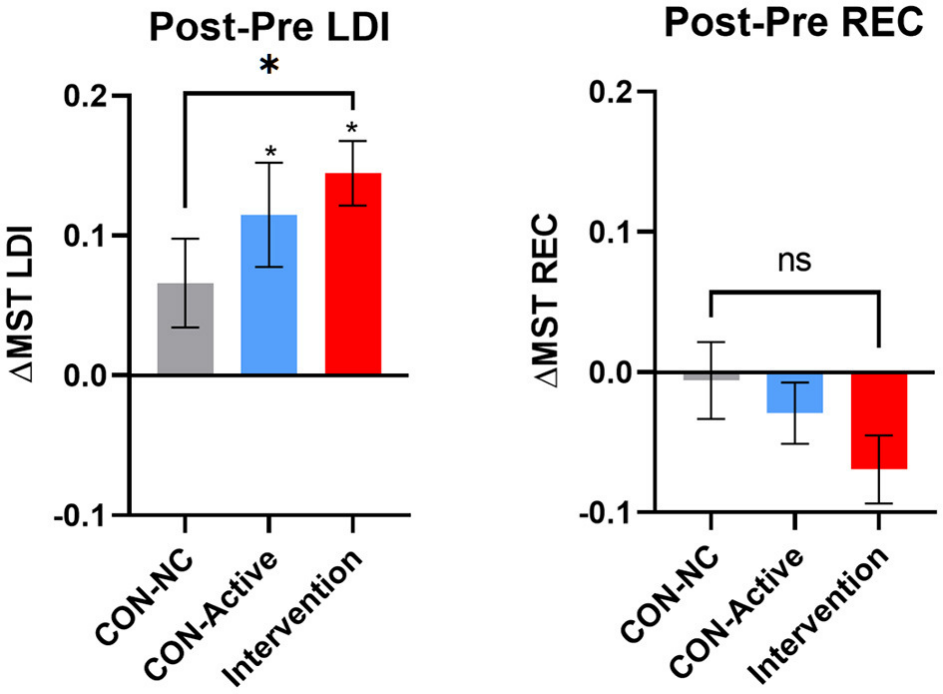
Change in the MST's LDI (left) and REC (right) measures for the no-contact control (CON-NC, gray), active control (CON-Active, blue), and intervention (red) groups. Error bars represent SEM. A reliable improvement in memory ability was found in the primary endpoint (LDI measure for CON-NC vs. Intervention, *p* < 0.05). In addition, in both the CON-Active and intervention conditions, the improvement in LDI was reliably above chance (*over individual bars, *p*'s < 0.01, corrected).

Our primary outcome measure was the difference in LDI following training for the Intervention group relative to the CON-NC group (Figure 2, left). Here, we found a reliably larger increase in LDI performance in the Intervention group [unpaired *t*-test *t*_(59)_ = 2.08, *p* < 0.05]. Secondary outcome measures, comparing the Intervention group to the CON-Active group showed this difference to be unreliable [unpaired *t*-test *t*_(50)_ = 1.2, *p* = 0.24] and likewise, an unreliable difference between the two control groups [unpaired *t*-test *t*_(45)_ = 0.7, *p* = 0.49], consistent with the CON-Active condition providing a modest benefit (an exploratory *post-hoc* analyses showed no reliable difference between the two sub-groups in our Intervention condition; *p* = 0.33). Further, both the CON-Active and the Intervention groups showed reliable pre-post increases in LDI [paired *t*-test *t*_(19)_ = 3.08, *p* < 0.01 and *t*_(32)_ = 6.24, *p* < 0.0001; using Bonferroni-corrected alpha threshold of *p* < 0.0167], correcting for multiple comparisons.

As anticipated, there was no effect on the REC scores on the MST (Figure 2, right). Here, there was a numerical decrease in REC scores in the Intervention group that was not reliable [*t*_(59)_ = 1.6, *p* = 0.12].

## Discussion

**Figure 3:**
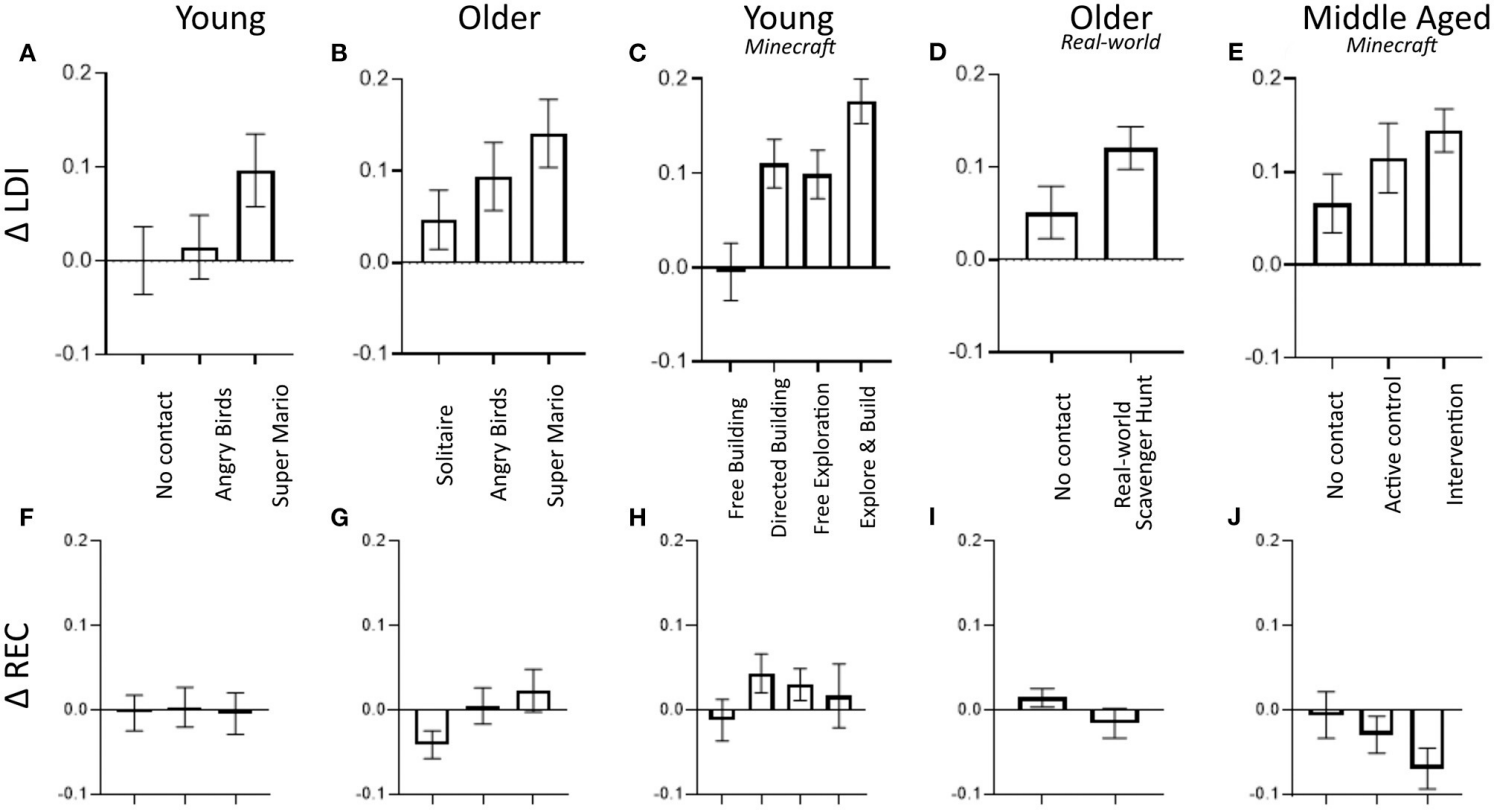
Results from four prior studies and the current study showing a consistent pattern of improvement in LDI scores (top) that tracks the amount of enrichment with no change in REC scores (bottom). Data from: **(A,F)** Clemenson and Stark (2015), **(B,G)** Clemenson et al. (2020), **(C,H)** Clemenson et al. (2019), **(D,I)** Kolarik et al. (2020), and **(E,J)** the present study. In each, conditions are ordered left to right in order of presumed level of enrichment or engagement.

The present study sought to determine whether playing *Minecraft* would improve memory function in middle-aged adults in the way it has improved memory function in younger adults (Clemenson et al., 2019) and in the way other video games centered on exploration and with large amounts of novelty have improved memory ability in both younger (Clemenson and Stark, 2015) and older (Clemenson et al., 2020; Wais et al., 2021) adults. The results were remarkably consistent with our prior work (Figure 3) and the work of others (Wais et al., 2021). Even modest amounts of gameplay (e.g., 30 min/day over 2–4 weeks) improved performance on a hippocampal dependent memory task while not affecting a simpler recognition memory task that is far less reliant upon the hippocampus. We should note that while long-term follow-up data were not available here, our prior studies have shown little decline when tested 2–8 weeks after the intervention (Clemenson and Stark, 2015; Clemenson et al., 2020; Kolarik et al., 2020), suggesting the effects are not terribly short-lived.

A summary of our studies exploring game play on memory performance is presented in Figure 3. In younger adults (18–22 years; Figures 3A,F), hippocampal-based memory improved following 2-weeks of training (30m/day) using *Super Mario 3D World* (right), with the amount of improvement correlating with the amount of exploration in the game (Clemenson and Stark, 2015). In contrast, a no-contact control group (left) and using the simpler game *Angry Birds* (middle) showed no effect (also demonstrating the lack of practice effects in the LDI measure). In older adults (60–80 years; Figures 3B,G), 1-month of playing *Super Mario 3D World* resulted in a robust improvement in our LDI measure, while playing *Solitaire* showed no effect (Clemenson et al., 2020). Here, playing *Angry Birds* (having a game console at home and learning to use it is a very novel experience for those in their 70's) resulted in an intermediate effect. In young adults, *Minecraft* (Figures 3C,H) allowed us to more directly manipulate the amount and type of enrichment (Clemenson et al., 2019). Our prior work found that 2-weeks of spatial exploration of the world and building complex structures with resources gathered in the world (Explore and Build; right) resulted in a robust improvement in LDI, unconstrained building in a world devoid of spatial features (Free Building, left) showed no effect on LDI, and having participants learn to build complex structures in a flat world (Directed Building, mid-left) or having them merely explore the world (Free Exploration; mid-right) both involved a discernable degree of enrichment and both resulted in strong effects (albeit seemingly less robust than the combination). In the results presented here (Figures 3E,J), we found identical effects in our Intervention group (right), that paralleled the Free Exploration and Directed Building groups from the young participant study. There, what was not enriching to young adults (Free Building) showed a modest effect in middle-aged participants (who were playing a novel immersive 3D game, albeit in a more spartan mode; middle bar), paralleling the improvement in *Angry Birds* found in older adults. Finally, we have investigated this effect outside the realm of computerized video games to a real-world enrichment paradigm in older adults (Kolarik et al., 2020). Here, adults performed a scavenger-hunt style game in a local park, learning the locations associated with 20 cues over the course of a week before shifting to another park in a 1-month intervention. As with the game-based interventions, performing this spatial learning task improved LDI scores with no effect on simple recognition memory (Figures 3D,I). Thus, in each study, there appears to be a comparable amount of improvement in the LDI as well as a relationship between the amount of enrichment or engagement and the improvement in the LDI (with the clear caveat that it is currently very difficult to measure this amount of enrichment or engagement).

We should note that these improvements are not trivial. The observed effect sizes shown in each of the studies in Figure 3 average a Cohen's d of 0.76 (range 0.51–1.48). In our prior work in healthy aging, we have found a very clear age-related decline (Stark et al., 2013) in the LDI of ~0.06 per decade (cross-sectionally measured). In the present study, the LDI improvement would amount to 13 years' worth of that decline and across all the studies, it averages the same as 17.3 years of decline (range 11.6–30.1). The size of the effect is remarkably consistent across all five studies.

Therefore, it seems reasonable to conclude that playing complex, off-the-shelf 3D video games can have positive effects on memory ability. There is other evidence that commercial video games can result in positive benefits in cognitive functioning in older adults (Belchior et al., 2016, 2019; Vázquez et al., 2018; Perrot et al., 2019), including on tasks of executive functioning, attention, and working memory. Similarly, video game training has been shown to enhance the neural signature of cognitive control using EEG in young adults (Anguera et al., 2013). Yet, there is also a burgeoning (and controversial) industry in “brain training” games, typically taking the form of puzzles or exercises that are designed to tap specific perceptual or cognitive processes (e.g., visual attention or working memory), but that often do not show the “far transfer” and ability to transfer in-game improvements to performance outside of the game (Nouchi et al., 2012; Makin, 2016; Melby-Lervåg et al., 2016; Simons et al., 2016). Both here, and in our prior work, the interventions have little, if any, direct relationship to the memory task being used as a primary outcome measure, demonstrating this transfer. In some cases, such as playing Angry Birds, it would appear as if remembering specific details of an image such as the configuration of your current attempt, would map more clearly onto the LDI metric. Yet, this style of game has proven less effective, despite the closer transfer potential.

We suggest that these are commercial video games plays heavily into our observed results. To succeed commercially, games must provide users with rich, engaging experiences that they are willing to pay for. While human studies have shown that the hippocampus plays an active role in the navigation and spatial memory of lab-based virtual environments (Maguire, 1998; Burgess et al., 2002; Ekstrom et al., 2003), the experiences in modern video games, created by large teams of professionals with multi-million dollar budgets, are simply far richer, engaging, and full of long-playing content than laboratory-derived games or the simple games found in many “brain training” exercises. In addition, in contrast to “brain training” games, commercial video games are not created with specific cognitive processes in mind, but rather are designed to captivate and immerse the user into characters and adventures. Rather than isolate single brain processes, video games can naturally draw on or require many cognitive processes including visual, spatial, emotional, motivational, attentional, critical thinking, problem solving, working memory, etc. It is quite possible that by explicitly avoiding a narrow focus on a single or small set of cognitive domains and by more closely paralleling natural experience, immersive video games may be better suited to provide enriching experiences that translate into functional gains. We note, however, that our proposed mechanisms are speculative at this point as direct studies of this have not been done. However, given the consistent results observed, comparing this style of game to other games or other cognitive interventions is clearly warranted.

Finally, we should note that playing these games mirrors real-world experiences and the richness of the games provides a clear parallel to environmental enrichment studies in the rodent. There, complex environments, rich experiences, and exercise are often combined to yield increase in adult neurogenesis (van Praag et al., 1999b), synaptogenesis (Rampon et al., 2000; Gogolla et al., 2009), density and complexity of dendrites (Moser et al., 1994; Rampon et al., 2000; Faherty et al., 2003; Zhao et al., 2014; Gonçalves et al., 2016), enhancing long-term potentiation (van Praag et al., 1999a; Farmer et al., 2004), and increasing expression of synaptic proteins (Nithianantharajah et al., 2004), all in the hippocampus. The fact that we have consistently observed improvements in memory tasks that require the hippocampus, but no effects outside of this form of memory bolsters the hypothesis that the effects observed here are related to the effects of enrichment found in rodents. In conclusion, video games provide an avenue for intervention and possibly rehabilitation, improving memory and other cognitive functions through the engagement of a diverse array of neurobiological mechanisms.

## Data Availability Statement

The raw data supporting the conclusions of this article will be made available by the authors, without undue reservation.

## Ethics Statement

The studies involving human participants were reviewed and approved by University of California, Institutional Review Board. The patients/participants provided their written informed consent to participate in this study.

## Author Contributions

The study was designed by CS, GC, and SS. The manuscript was initially written by CS. All authors participated in data collection, data analysis, and contributed to its revisions.

## Conflict of Interest

The authors declare that the research was conducted in the absence of any commercial or financial relationships that could be construed as a potential conflict of interest.
